# Case of Removal of the Superior Maxillary Bone for an Encephaloid Tumor

**Published:** 1855-03

**Authors:** H. A. Potter

**Affiliations:** Geneva, N. Y.


ART. III.—*Case of Removal of the Superior Maxillary Bone for an Encephaloid Tumor.* By H. A. POTTER, M. D., Geneva, N. Y.

The subject of this operation is a Mrs. Ensley, aged 54, a strong athletic woman, the wife of a wealthy farmer, and accustomed to hard labor. No malignant disease of any kind has ever been known in the family. Mrs. Ensley had received two injuries, within the last eighteen months, upon the side of the head and face now involved in the disease, and produced much effusion about the eye and face; but she does not know as either was directly on the face or over the antrum where the tumor originated, but thinks they were about the side of the head and angle of the eye; she has had much pain about the head since that time.

The end of April or the early part of May last, she first began to feel pain about the region of the antrum of the left side in the superior maxillary bone; this gradually increased, and soon a slight discharge appeared from the nostril of that side, of a watery character as she described it; this continued with increased pain until the 7th of August, when I was first consulted in the case. I found a polypus in the left nostril and a slight general enlargement and thickening of the integuments of the whole side of the face. I removed the polypus; apprised them of the approaching danger; said to them I thought as the disease originated in the antrum the polypus would return. I saw nothing more of her until October, when the polypus was being reproduced, and now the entire face on the left side was fairly involved in one general tumor. I gave her no encouragement with or without an operation. She suffered much pain, but still had hopes. The discharge from the nostril was somewhat increased. She now consulted quacks, took their medicine, and had some hopes of being cured. I saw no more of her until the early part of December, when she came to my office in despair; her pain had become almost unendurable; the tumor much enlarged; the teeth, from the eye tooth back, all were loosened; and on raising the upper lip a general thickened mass, purple in appearance, with a hard feel, involved the whole extent of jaw and alveolar process. I only confirmed what I had previously stated. She now felt she must have something done; still she consulted other physicians and was advised not to have the tumor removed. Her husband was absent from home for a period of time; he was recalled and at once came for me to visit his wife. I accordingly complied, and on the same afternoon I took my young friend, Dr. Bowen, Professor of Anatomy in Geneva Medical College, and visited the patient. We examined the case, and after consulting and leaving all the responsibility upon themselves, and they seeing there was no chance without an operation, and but a faint hope with one, yet as it was the only possible chance, we advised the operation, and the next day but one we, in company with Professors Charles A. Lee, Hawley, Bowen, and other physicians, repaired to the house; all things being ready, the operation was performed in the following manner:

Chloroform being administered, the incision through the integuments was commenced anterior and superior to the ear, and then along a little downward and forward along the inferior margin of the zygomatic arch, and h en forward over the superior portion of the malar bone and a little beneath the inferior border of the inferior orbital plate, and in a little curved direction to near the nasal plate, and completed the incision at the angle of the mouth, which point will be found to vary but a slight angle from the place of commencing the operation.

By this incision all the important parts were avoided; the nerves, blood-vessels, and salivary duct were all left entire in the flap, and no assistant required to keep it from troubling the operator. The difficulty, as anticipated, was found to commence in the antrum, and the entire bones of the left side of the face were found involved in one grand mass of disease; the superior maxillary, the malar, and a portion of the temporal were removed; all of the maxillary, except a portion forming the socket; nearly all the inferior border forming the inferior portion of the bony orbit; all the antrum and the bone were removed from their connections with the plerygoid plates of the sphenoid bone, and I also removed all the malar bone except a small portion forming the outer angle of the eye and a portion of the temporal forming the zygomatic process; the flaps were then adjusted, the wound healed by the first intention, the cavity filled up with healthy granulations, and this, the twenty-second day from the operation, there is no appearance of a return of the disease. The patient is discharged and about the house.

All the teeth from behind forward to the eye tooth, were loose, and of course with the bone removed.

GENEVA, N. Y., January 20, 1855.

